# Determination of the absolute oral bioavailability of niraparib by simultaneous administration of a ^14^C-microtracer and therapeutic dose in cancer patients

**DOI:** 10.1007/s00280-017-3455-x

**Published:** 2017-10-17

**Authors:** L. van Andel, H. Rosing, Z. Zhang, L. Hughes, V. Kansra, M. Sanghvi, M. M. Tibben, A. Gebretensae, J. H. M. Schellens, J. H. Beijnen

**Affiliations:** 1 Department of Pharmacy and Pharmacology, Antoni van Leeuwenhoek/The Netherlands Cancer Institute and MC Slotervaart, PO Box 90440, 1006 BK Amsterdam, The Netherlands; 2 TESARO, Inc., Waltham, MA USA; 3 Xceleron, Inc., A Pharmaron Company, Germantown, MD USA; 4 Division of Clinical Pharmacology, Department of Medical Oncology, The Netherlands Cancer Institute, Amsterdam, The Netherlands; 5 Division of Pharmacoepidemiology and Clinical Pharmacology, Faculty of Science, Department of Pharmaceutical Sciences, Utrecht University, Utrecht, The Netherlands

**Keywords:** Niraparib, Bioavailability, AMS, LC–MS/MS, Pharmacokinetics, ^14^C-microtracer

## Abstract

**Introduction:**

Niraparib (Zejula™) is a poly(ADP-ribose) polymerase inhibitor recently approved by the US Food and Drug Administration for the maintenance treatment of patients with recurrent platinum-sensitive epithelial ovarian, fallopian tube, or primary peritoneal cancer who are in a complete or partial response to platinum-based chemotherapy. The pivotal phase III clinical trial has shown improved progression-free survival in patients receiving niraparib compared with those receiving placebo.

**Purpose:**

Since niraparib is administered orally, it is of interest to investigate the oral bioavailability (*F*_po_) of this novel compound, which is the aim of this study.

**Methods:**

Six patients received an oral therapeutic dose of 300 mg niraparib, followed by a 15-min intravenous infusion of 100 µg ^14^C-niraparib with a radioactivity of approximately 100 nCi. The niraparib therapeutic dose was measured in plasma using a validated liquid chromatography–tandem mass spectrometry method, whereas the total ^14^C-radioactivity and ^14^C-niraparib plasma levels were measured by accelerator mass spectrometry and a validated high performance liquid chromatography assay with AMS.

**Results:**

The *F*_po_ of niraparib was determined to be 72.7% in humans.

## Introduction

Niraparib (Zejula™) is a novel poly(ADP-ribose) polymerase (PARP) inhibitor that has recently been approved by the US Food and Drug Administration (FDA) for the maintenance treatment of patients with recurrent, platinum-sensitive epithelial ovarian, fallopian tube, or primary peritoneal cancer who are in a complete or partial response to platinum-based chemotherapy. The recommended starting dose of niraparib has been established at 300 mg once daily in the form of three capsules of 100 mg each [1]. Mirza et al. have recently published the results of a large phase III study in patients with recurrent, platinum-sensitive ovarian cancer [2]. The main conclusion was that progression-free survival was significantly longer in patients receiving niraparib than in those receiving placebo.

Since niraparib is administered orally it is of interest to investigate the fraction of the oral dose that reaches systemic circulation unchanged, referred to as the drug’s oral bioavailability (*F*_po_). The FDA has described its recommendations regarding the determination of the bioavailability of a new drug product [3]. To determine the bioavailability of an orally administered drug, a systemic exposure profile should be obtained by measuring drug concentrations in systemic circulation (i.e., in plasma). Bioavailability studies assess the performance of the formulation, and the results from these trials can be used as a reference for subsequent bioequivalence studies. One of the most common reasons for drug development failure is a drug formulation’s poor bioavailability; hence, bioavailability should be investigated as early as possible during drug development [4]. Low oral bioavailability can be caused by different factors, such as a drug’s inability to cross cell membranes, poor water solubility when a drug is administered as a solid, and metabolic instability [5, 6]. Moreover, it has been observed that low bioavailability of a drug is associated with increased variability in individual exposure levels and inconsistent performance. This intersubject variability can be clinically significant when prescribing products with low bioavailability, especially when these drugs have a narrow therapeutic window and increased risk of toxicity [7].

Pharmacokinetic studies with niraparib in Sprague–Dawley rats and beagle dogs yielded *F*_po_ values of 27 and 57%, respectively (data not published). However, the bioavailability of niraparib in humans is still unknown. This study determined the bioavailability of niraparib in patients with advanced cancer by simultaneously dosing cancer patients with an orally therapeutic dose and an intravenous (IV) ^14^C-microtracer—an approach extensively described by Lappin and colleagues [5, 8]. The term microtracer is used to refer to the intravenous dose of an isotopically labelled drug in these absolute bioavailability studies and is typically ≤ 1% of the therapeutic dose [8]. The radioactive microtracer technique has increasingly replaced the traditional crossover study, in which patients receive a non-IV dose and an IV dose in separate periods with a washout period in between. The microtracer approach has several advantages: the development of a stable IV formulation of the drug is not necessary and the accompanying toxicology studies are not required [9–11]. Moreover, even poorly soluble drug substances are often easily formulated as solution for IV at such low doses [5, 10], and problems with nonequivalent clearance between the IV and non-IV doses in the traditional crossover design are eliminated [9].

In microtracer bioavailability studies, the radiolabel allows researchers to distinguish the IV dose and the extravascular dose. Additionally, the microtracer dose does not significantly contribute to systemic drug concentrations arising from the oral dose [8]. The therapeutic dose-plasma concentrations are analysed by a validated liquid chromatography–tandem mass spectrometry (LC–MS/MS) assay, and the ^14^C-microtracer levels are determined by accelerator mass spectrometry (AMS). The high sensitivity of AMS allows the determination of very low analyte concentrations, making it possible to give patients an extremely low radioactive dose that does not require regulatory approval or dosimetry studies [5, 9]. Another advantage of AMS is that the extravascular and IV doses can be administered simultaneously, so there is no need for a crossover study, considerably shortening the experimental phase and reducing the burden and risk for the patient. Moreover, the potential problem of intrapatient variability observed in crossover studies is eliminated. Since the ^14^C-microtracer is administered intravenously, 100% of the microdose enters systemic circulation. Comparing the dose-normalised area under the curves (AUCs) of the therapeutic dose and the ^14^C-microtracer allows the calculation of oral bioavailability. This approach has been used previously to determine the absolute bioavailability of a variety of drugs across a range of therapeutic areas [12–14]. In addition, total ^14^C-radioactivity was measured by AMS to determine the extent of metabolic burden.

## Materials and methods

### Clinical study design

**Fig. 1:**
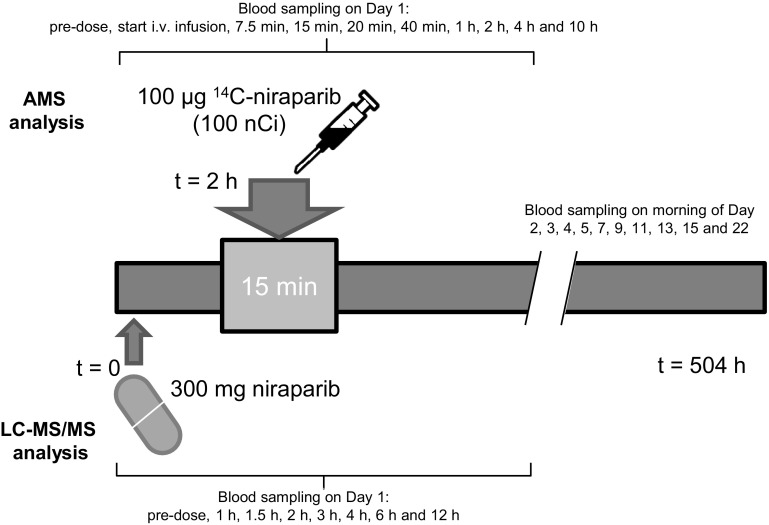
Study design

**Fig. 2:**
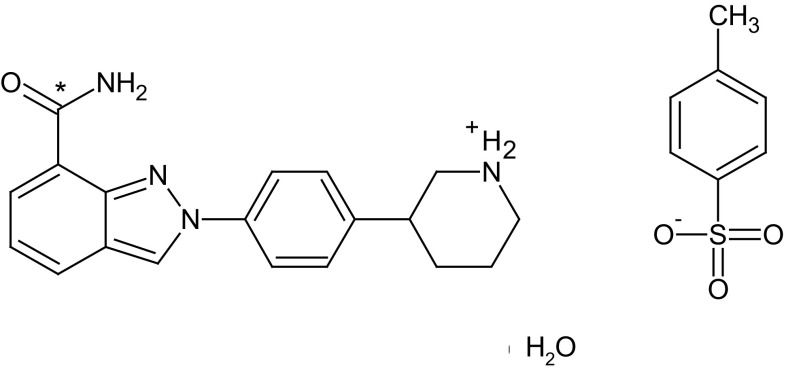
Molecular structure of ^14^C-niraparib tosylate salt. The asterisk denotes the position of the radioactive label in the IV formulation

Six evaluable patients were included in the clinical trial (NCT02476552). Signed and dated written informed consent was obtained from each patient before participation in the study and before the performance of any procedures. After informed consent was obtained, eligible participants were given the choice to be admitted to the clinical research unit on day − 1 or on day 1. A summary of the study design can be found in Fig. 1. After an overnight fast of at least 10 h (h), subjects received an unlabelled oral dose of 300 mg niraparib [three capsules of 100 mg, supplied as the tosylate salt (Almac, Craigavon, UK); the same formulation as the currently marketed Zejula™; Fig. 2], after which they received a 15-minute (min) IV infusion of 100 µg niraparib labelled with ^14^C (Quotient Clinical, Ruddington, Nottingham, UK). Based on the anticipated time maximum plasma concentration (*C*_max_) was reached (*t*_max_), the IV infusion was administered 2 h after the oral dose. Subjects continued fasting until 2 h after the start of the IV infusion, at which time a non-standardised light meal was served. The radiolabelled dose contained approximately 100 nCi (3.7 kBq) radioactivity and was administered into one arm of the subject; the contralateral arm was used for pharmacokinetic sampling and clinical laboratory blood draws. The syringe for IV drug administration was connected to a Perfusor Space Infusion Pump (B. Braun), which was set to administer 7 mL in 15 min. Blood samples were taken pre-dose and at 1, 1.5, 2 (just before start of IV infusion), 2.125 (halfway through the IV infusion), 2.25 (just before end of infusion), 2.33, 2.66, 3, 4, 6, and 12 h. Sampling was done on each morning of days 2, 3, 4, 5, 7, 9, 11, 13, 15, and 22. Plasma was divided in two sets: one set was sent to Xceleron Inc. (Germantown, MD, USA) for the determination of plasma concentrations of the ^14^C-microtracer by AMS, and the other set was stored at the Antoni van Leeuwenhoek Hospital until it received pharmacokinetic analysis at the Good Laboratory Practice–licensed laboratory of the pharmacy. Plasma was separated from blood by centrifugation (3,000 rpm, 15 min, 4 °C) and was stored frozen at ≤ − 70 °C in polypropylene tubes before and after analysis. After completion of the study, all participants entered an extension study of niraparib (with no washout period required) and continued receiving niraparib on a daily basis for 4 weeks. Vital signs assessments, haematology assessment, and blood chemistry assessments were done frequently to make sure that the patients were still in sufficient condition to remain on treatment. Based on these results, clinicians were allowed to reduce the dose twice by one capsule, if necessary. The study was approved by the local independent ethics committee and was conducted in accordance with local regulations, the Declaration of Helsinki, and International Conference on Harmonisation Good Clinical Practice Guidelines.

### Patients

Patients over the age of 18 who might benefit from treatment with a PARP inhibitor were included in the study. Patients with a histologically or cytologically confirmed diagnosis of metastatic or locally advanced solid tumours who had failed to respond to standard therapy, who had progressed despite standard therapy, or for whom no standard therapy existed were eligible for the study. Inclusion criteria included adequate organ function, such as bone marrow function, adequate renal function, and adequate hepatic function (absolute neutrophil count ≥ 1500/µL, platelets ≥ 100,000/µL, and haemoglobin ≥ 9 g/dL); serum creatinine ≤ 1.5 × upper limit of normal (ULN) or a calculated creatinine clearance ≥ 60 mL/min; total bilirubin ≤ 1.5 × ULN or direct bilirubin ≤ 1 × ULN; and alanine aminotransferase and aspartate aminotransferase ≤ 2.5 × ULN (or ≤ 5 × ULN in case of liver metastases). Only patients with Eastern Cooperative Oncology Group performance status of 0–2 were included in the study. Furthermore, patients were instructed to use effective forms of contraception. Patients were excluded from the study if they had undergone palliative radiotherapy within a week of the start of the trial; had at least grade 2 toxicities from prior cancer therapy; had brain or leptomeningeal metastases; had undergone major surgery within 3 weeks before start of the study; had any other serious, uncontrolled medical disorder; or had received a platelet transfusion within 4 weeks of niraparib administration. Immunocompromised patients as well as patients with confirmed or suspected hepatitis B virus, hepatitis C virus, or HIV were also excluded from the study. QTc prolonging medication, proton pump inhibitors, and H2 blockers were prohibited for the duration of the study.

### Administered dose

The syringe was weighed before and after administration to enable calculation of the exact amount of solution and radioactive dose given to each patient. Aliquots were taken from the original vial supplied by Quotient Clinical and placed in separate scintillation vials, which were weighed before and after dispensing. A volume of 10 mL of Ultima Gold scintillation cocktail (PerkinElmer, Groningen, The Netherlands) was added and the aliquots were measured for total radioactivity by liquid scintillation counting. Results were expressed in disintegrations per min, which were ultimately converted to total administered dose by multiplying by the total weight injected.

### Quantification of niraparib

Concentrations of niraparib were determined in plasma by analysis according to a validated LC–MS/MS method [15]. In brief, separation was carried out using an HPLC Acquity I Class pump (Waters, Milford, MA, USA) supplied with a SunFire C18 column (50 mm × 2.1 mm, 5 µm, Waters). Gradient elution was applied using 20 mM ammonium acetate in water (mobile phase A) and 0.1% formic acid in acetonitrile-methanol (50:50, v/v; mobile phase B). Each batch of experimental samples was run against freshly prepared calibration standards. Replicate quality control samples at three concentrations were stored and analysed alongside study samples. The analytical range was from 1 to 500 ng/mL. The concentrations of niraparib were reported using Analyst 5.2, Sciex, Framingham, MA, USA software.

### ^14^C-niraparib and total ^14^C-radioactivity analysis by accelerator mass spectrometry

Total ^14^C-radioactivity concentrations in plasma samples were determined using AMS. Plasma samples were directly aliquoted into tubes containing copper oxide powder (CuO). The samples were graphitised and analysed by a Single Stage Accelerator Mass Spectrometer (SSAMS) 250 kV system (National Electrostatics Corp., Middleton, WI, USA). Plasma concentrations for the administered ^14^C-niraparib were determined by a validated HPLC + AMS method by Xceleron Inc. Plasma sample pre-treatment involved protein precipitation using acetonitrile, after which samples were vortex-mixed and centrifuged (10 min, 3,750 rpm, 4 °C) and the clean supernatant was evaporated to dryness under a gentle stream of nitrogen. Samples were analysed by an Agilent 1200 HPLC system (Agilent Technologies, Palo Alto, CA, USA) equipped with a wavelength UV detector set to 254 nm. Separation was achieved using a Phenomenex Kinetex 2.6 µ biphenyl column (100 Å, 100 × 3.0 mm). Mobile phase A and B consisted of 20 mM ammonium acetate in water and 0.1% formic acid in acetonitrile–methanol (50:50, v/v), respectively. Column oven temperature was set at 40 °C, and run-time was 18 min. Unlabelled niraparib was used as an internal standard. Eluate was collected using a fraction collector, and resulting fractions containing ^14^C-niraparib were transferred into tubes containing baked CuO powder and sodium benzoate. The fractions were graphitised and analysed by SSAMS. Each batch of experimental samples was run against freshly prepared calibration standards at seven concentrations in duplicates. Replicate quality control samples at three concentrations were stored and also analysed alongside study samples. Finally, *F*_po_ was calculated as the ratio of the dose-normalised AUC of the oral dose to the IV dose.

$${F_{{\text{po}}}}\left( \% \right){\text{ }}={\text{ 100\% }} \times \frac{{({\text{AU}}{{\text{C}}_{0{\text{-inf }}}}\,{\text{of niraparib from oral dose/3}}00{\text{ mg}})}}{{\left( {{\text{AU}}{{\text{C}}_{0{\text{-inf }}}}\,{\text{o}}{{\text{f}}^{{\text{ 14}}}}{\text{C-niraparib from IV dose/actual IV dose}}} \right)}}~$$

### Pharmacokinetic analysis

Plasma niraparib and radioactivity concentrations were used to determine the following pharmacokinetic parameters: *C*_max_; time to reach *C*_max_ (*t*_max_); AUC from time 0 to the last quantifiable concentration (AUC_0–last_; calculated using the linear-up/log-down trapezoidal rule, with the linear trapezoidal rule applied up to the *t*_max_ and the log-linear trapezoidal rule applied after the *t*_max_); AUC from time 0 to infinity (AUC_0–inf_; calculated using the linear-up/log-down trapezoidal rule); volume of distribution (*V*_d_); apparent oral volume of distribution (*V*_d_/*F*); clearance (CL); apparent oral clearance (CL/*F*); and half-life (*t*_½_). Analyses were done by a noncompartmental method using WinNonlin Phoenix Version 6.2.1 or higher (Pharsight Corporation, St. Louis, Missouri, USA). All calculations for final analysis were based on actual sampling times.

## Results

### Patients

Six female patients participated in the clinical study, four with breast cancer, one with ovarian cancer, and one with fallopian tube cancer (Table 1). All six patients who entered screening completed the study and were eligible for continued treatment with niraparib in the extension trial.

**Table 1:** Patient characteristics at baseline

Parameter	Value
Age (years)	
*n*	6
Mean (range)	51.5 (33–71)
Gender, *n* (%), female	6 (100)
Weight (kg)	
*n*	6
Mean (SD)	66.7 (11.5)
Tumour type	
Breast cancer	4 (66.7)
Ovarian cancer	1 (16.7)
Fallopian tube cancer	1 (16.7)

*SD* standard deviation

### Administered dose

Table 2 shows the administered dose and administered radioactivity for each patient. The measured ^14^C-niraparib dose was between 80.1 and 104.3 µg, and the administered radioactivity was equal to or less than 100 nCi (67–101) for each patient.

**Table 2:** Administered IV niraparib dose and radioactivity dose (*n* = 6)

	Administered dose (µg)	Administered radioactivity (nCi)
Patient number		
001	104.3	82
002	95.8	67
003	98.5	80
004	97.2	91
005	80.1	76
006	100.1	101
Mean (SD) [CV (%)]	96 (8.3) [9]	82.8 (11.9) [14]

*CV* coefficient of variation, *SD* standard deviation

### Pharmacokinetic analysis

**Fig. 3:**
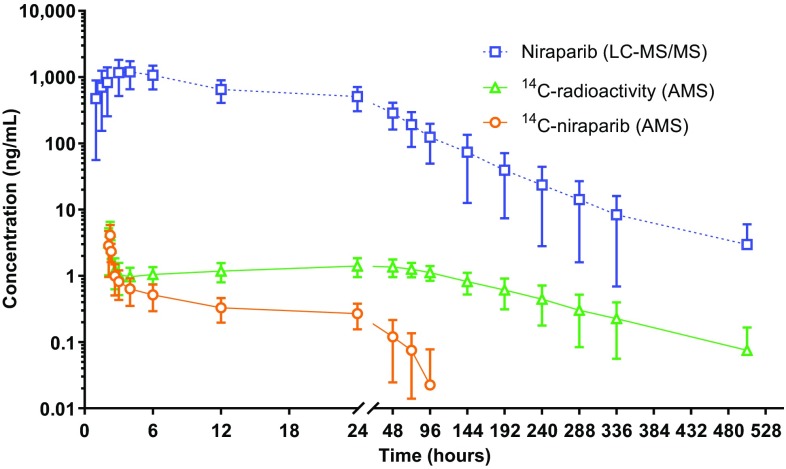
Mean (± SD) log-linear plasma concentration–time profiles of ^14^C-radioactivity, ^14^C-niraparib, and unlabelled niraparib in patients with advanced cancer (*n* = 6). ^14^C-niraparib and total ^14^C-radioactivity levels are expressed as ng-equivalents/mL. The ^14^C-dose is administered 2 h after the oral dose. Three down error bars are not shown avoiding the inappropriate negative value. *AMS* accelerated mass spectrometry, *LC–MS/MS* liquid chromatography–tandem mass spectrometry

Figure 3 shows plasma concentration–time curves for ^14^C-radioactivity, ^14^C-niraparib, and unlabelled niraparib.

### Pharmacokinetics of unlabelled niraparib

Unlabelled niraparib was rapidly absorbed, peaking 4 h (range 3.07–6.03) post-dose, reaching a *C*_max_ of 1,270 ng/mL. Elimination of the therapeutic dose was slow, with mean *t*_½_ of 95.6 h. *V*_d_/*F* and CL/*F* was 1.220 and 8.39 L h, respectively. Interpatient variability was moderate to high, with coefficient of variation (CV) ranging from 16.9 to 91.3%.

### Pharmacokinetics of ^14^C-radioactivity

^14^C-radioactivity in plasma could be measured up to 504 h after dosing for all six patients. Total ^14^C-radioactivity concentrations peaked to 4.65 ng-equivalents/mL at the end of the infusion. After reaching *C*_max_ at 0.25 h, coinciding with the end of the 15 min infusion time, the plasma concentrations of total ^14^C-radioactivity were slowly eliminated in a biphasic manner. Mean *t*_½_ was long and CL and *V*_d_ were low. The intersubject variability of pharmacokinetic parameters was moderate, with CV ranging from 21.2 to 51.2%.

### Pharmacokinetics of ^14^C-niraparib

Plasma levels of ^14^C-niraparib could not be detected with AMS after the 96-h time point. Concentrations peaked at 4.19 ng-equivalents/mL at the end of the infusion. After reaching *C*_max_ at 0.25 h, the plasma concentrations of ^14^C-niraparib slowly eliminated in a biphasic manner. The mean *t*_½_ was 28.2 h. The *C*_max_ and AUC_0–inf_ of the ^14^C-niraparib dose were 303-fold and 2,205-fold lower, respectively, than those of the unlabelled niraparib dose. The mean CL of ^14^C-niraparib was 5.90 L h and the *V*_d_ was 194 L, indicating low clearance and large volume of distribution with potential high niraparib tissue penetration. Intersubject variability was moderate, with CV ranging from 22.1 to 64.1%.

### Bioavailability

Figure 3 shows the plasma concentration–time curves for niraparib both from the radiolabelled microtracer, as measured by AMS, and from the therapeutic dose of 300 mg, as measured by LC–MS/MS. Unlabelled niraparib concentrations are expressed in ng/mL, whereas ^14^C-niraparib levels are expressed as ng-equivalents/mL. Niraparib *F*_po_ was assessed by comparing dose-normalised AUC_0–inf_ following oral administration, with dose-normalised AUC_0–inf_ of IV ^14^C-niraparib. Statistical analysis of pharmacokinetic parameters using analysis of variance to assess niraparib *F*_po_ is shown in Table 3. *F*_po_ of niraparib based on dose-normalised AUC_0–inf_ was determined to be 72.7% (90% confidence interval (CI): 61.69, 83.40).

**Table 3:** Summary of pharmacokinetic parameters of unlabelled niraparib, ^14^C-niraparib, and total ^14^C-radioactivity (*n* = 6)

PK parameter^a^	Total ^14^C-radioactivity^c^	100 µg IV ^14^C-niraparib^c^	Oral 300 mg niraparib
*C*_max_ (ng/mL)	4.65 (44.6)	4.19 (45.0)	1270 (48.1)
*t*_max_ (h)^b^	0.25 (0.12–0.25)	0.25 (0.12–0.25)	4.01 (3.07–6.03)
AUC_0–last_ (ng × h/mL)	276 (30.8)	16.4 (53.1)	46,900 (43.8)
AUC_0–inf_ (ng × h/mL)	296 (32.1)	21.5 (49.3)	47,400 (44.2)
*t*_½_ (h)	96.0 (35.8)	28.2 (43.9)	95.6 (16.9)
*V*_d_ and *V*_d_/*F* (L)	45.7 (21.2)	194 (22.1)	1220 (91.3)
CL and CL/*F* (L/h)	0.372 (51.2)	5.90 (64.1)	8.39 (73.8)
*F*_po_ (%) [90% CI]^d^	NA	NA	72.7 (18.2) [61.69,83.40]

*AUC*_*0–inf*_ area under the plasma concentration–time curve extrapolated to infinity, *AUC*_*0–last*_ area under the plasma concentration–time curve from time 0 to the last quantifiable concentration, *CI* confidence interval, *CL* clearance, *CL/F* apparent oral clearance, *C*_*max*_ maximum observed plasma concentration, *CV* coefficient of variation, *F*_*po*_ = absolute oral bioavailability, *IV* intravenous, *NA* not applicable, *PK* pharmacokinetic, *t*_½_ terminal half-life, *t*_*max*_ time to reach maximum observed plasma concentration, *V*_d_ volume of distribution, *V*_d_/*F* = apparent oral volume of distribution

^a^For all parameters, mean ± SD is presented

^b^ *t*_max_ is relative to the start of IV infusion. For *t*_max_, median and range are presented

^c^For ^14^C-radioactivity and ^14^C-niraparib, *C*_max_ and AUC are presented in ng-equivalents/mL and ng-equivalents × h/mL, respectively

^d^Model-based (least squares) geometric mean: analysis of variance extracting the effects due to treatment and subject

## Discussion

The purpose of the present study was to assess the bioavailability of niraparib administered at the suggested starting dose of 300 mg. We employed the technique of simultaneous dosing of the oral formulation and the IV ^14^C-microtracer, which has advantages over the traditional crossover study design, most importantly diminished burden to the patient and the extremely low radioactive dose. In addition, intrapatient variability is minimised by administering both doses within the same time period.

As expected, the difference between the AUC_0–inf_ of unlabelled niraparib and ^14^C-niraparib was more than 2000-fold, which is generally consistent with a microtracer concentration 3000-fold lower than the therapeutic dose (300 mg versus 100 µg). ^14^C-niraparib was detected by AMS up to 96 h post-dose and total ^14^C-radioactivity up to 504 h post-dose. The differences observed between the AUC of ^14^C-niraparib and total ^14^C-radioactivity is explained by niraparib-derived metabolite formation. This difference was more than tenfold, indicating that niraparib is extensively metabolised.

The *t*_½_ of total ^14^C-radioactivity was longer than the *t*_½_ of ^14^C-niraparib, which further confirms metabolite formation. Metabolites have been identified and quantified previously [16]. Besides niraparib, the main moiety in circulation, the amide-hydrolysed niraparib metabolite (M1) and its glucuronide (M10) were also substantially present in circulation. The slight increase seen between 4 and 48 h post-dose could indicate tissue distribution and redistribution into the plasma compartment. Moreover, the large *V*_d_/*F* and low CL/*F* observed indicates that niraparib is extensively distributed into the tissues.

After oral administration, niraparib was rapidly absorbed and slowly eliminated; the median *t*_max_ was 4.01 h and the mean *t*_½_ was approximately 95.6 h. The median *t*_max_ of unlabelled niraparib did not fully coincide with the IV infusion as intended. In a previous phase I dose-escalation study, the *t*_max_ of niraparib was around 3.1 h, with a range of 1.5–4.1 h [1]. The range and median in this study differed slightly, with a median *t*_max_ of approximately 4 h and a range of 3.07–6.03 h, but are consistent with the overall findings of the dose-escalation study.

As expected in this small group of patients, the intersubject variability of total ^14^C-radioactivity and ^14^C-niraparib-related pharmacokinetic parameters was moderate, with CV ranging from 21.2 to 51.2% and from 22.1 to 64.1%, respectively. Intersubject variability of unlabelled niraparib pharmacokinetic parameters was moderate to high, with CV% ranging from 16.9 to 91.3%. Subjects in the current study underwent a longer sampling period (22 days) than patients in a clinical trial published previously [1], allowing for a more complete coverage of the terminal phase for *t*_½_ determination. The authors suggest that the longer sampling period largely accounts for the discrepancy in *t*_½_ (36.2 h in the previous study versus 95.6 h in the present study).

It would be reasonable to believe that niraparib undergoes first-pass metabolism before reaching systemic circulation, since it is biotransformed in the liver to M1 which is subsequently conjugated with glucuronic acid to the glucuronide metabolite (M10). Therefore, metabolism by the liver could be attributed to the bioavailability of less than 100%. Nonetheless, such an effect is evidently modest, given the high bioavailability determined in this study.

Moreover, based on PK results, it can be concluded that niraparib is a low hepatic extraction drug. ^14^C-niraparib CL was low (5.9 L h), which means that, assuming that hepatic blood flow is approximately 80 L/h [17], the hepatic extraction ratio is very low. One could therefore expect that first-pass effects should not limit the oral bioavailability to a great extent.

A mass balance study in humans using radiolabelled niraparib investigated the amount of niraparib and niraparib-related compounds excreted in urine and in faeces [16]. Of the total dose orally administered, approximately 90% was recovered in excreta, with approximately 20% recovered as the parent compound in faeces. This portion of the oral dose was mostly excreted unchanged from the hepatobiliary route. These elimination findings were consistent, with an *F*_po_ of 72.7% and thus, the high *F*_po_ seen for niraparib is consistent with high intestinal absorption of the compound.

The results from this study, including the fact that niraparib has a high bioavailability along with low clearance and the long half-life has provided the rationale for a lower and less frequent dosing schedule in the clinic relative to other PARP inhibitors on the market and/or in development. Moreover, while the intersubject variability in exposure to niraparib appears to be moderate to high, such a variability would be unlikely due largely to the absorption of niraparib.

In summary, six patients received an oral therapeutic dose of 300 mg niraparib, followed by a 15-min IV infusion of 100 µg ^14^C-niraparib with a radioactivity of approximately 100 nCi. This resulted in an oral bioavailability of 72.7% (90% CI: 61.69,83.40) in cancer patients.
