# Correction to: Determination of the absolute oral bioavailability of niraparib by simultaneous administration of a ^14^C-microtracer and therapeutic dose in cancer patients

**DOI:** 10.1007/s00280-017-3474-7

**Published:** 2017-11-27

**Authors:** L. van Andel, H. Rosing, Z. Zhang, L. Hughes, V. Kansra, M. Sanghvi, M. M. Tibben, A. Gebretensae, J. H. M. Schellens, J. H. Beijnen

**Affiliations:** 1 Department of Pharmacy and Pharmacology, Antoni van Leeuwenhoek/The Netherlands Cancer Institute and MC Slotervaart, PO Box 90440, 1006 BK Amsterdam, The Netherlands; 2 TESARO, Inc., Waltham, MA USA; 3 Xceleron, Inc., A Pharmaron Company, Germantown, MD USA; 4 Division of Clinical Pharmacology, Department of Medical Oncology, The Netherlands Cancer Institute, Amsterdam, The Netherlands; 5 Division of Pharmacoepidemiology and Clinical Pharmacology, Faculty of Science, Department of Pharmaceutical Sciences, Utrecht University, Utrecht, The Netherlands

## Correction to: Cancer Chemother Pharmacol 10.1007/s00280-017-3455-x

The article “Determination of the absolute oral bioavailability of niraparib by simultaneous administration of a ^14^C-microtracer and therapeutic dose in cancer patients”, written by L. van Andel, H. Rosing, Z. Zhang, L. Hughes, V. Kansra, M. Sanghvi, M. M. Tibben, A. Gebretensae, J. H. M. Schellens and J. H. Beijnen, was originally published electronically on the publisher’s internet portal (currently SpringerLink) on 17th October 2017 without open access.

With the author(s)’ decision to opt for Open Choice the copyright of the article changed on 24th November to _ The Author(s) 2017 and the article is forthwith distributed under the terms of the Creative Commons Attribution 4.0 International License (http://creativecommons.org/licenses/by/4.0/), which permits use, duplication, adaptation, distribution and reproduction in any medium or format, as long as you give appropriate credit to the original author(s) and the source, provide a link to the Creative Commons license, and indicate if changes were made. The original article was corrected.

